# Prior pregnancy complications and maternal cardiovascular disease in young Korean women within 10 years after pregnancy

**DOI:** 10.1186/s12884-022-04578-2

**Published:** 2022-03-21

**Authors:** Geum Joon Cho, Ji Soo Um, Sa Jin Kim, Sung Won Han, Soo Bin Lee, Min-Jeong Oh, Jae Eun Shin

**Affiliations:** 1 Department of Obstetrics and Gynecology, College of Medicine, Korea University, Seoul, Republic of Korea; 2 Department of Obstetrics and Gynecology, College of Medicine, Bucheon St. Mary’s Hospital, The Catholic University of Korea, 327, Sosa-ro, Wonmi-gu, Bucheon-si, Gyeonggi-do 14647, Seoul, Republic of Korea; 3 School of Industrial Management Engineering, Korea University, Seoul, Republic of Korea

**Keywords:** Cardiovascular disease, Low birth weight, Pregnancy complication, Preeclampsia, Preterm delivery, Stroke

## Abstract

**Background:**

This study aimed to compare obstetric outcomes in Korean women with and without future cardiovascular disease (CVD) within 10 years after pregnancy, and assessed whether pregnancy complications are independent risk factors, and whether the combination of pregnancy complications has an additive function for risk factors for CVD.

**Methods:**

This was a nationwide population-based study combining the database of the Korea National Health Insurance claims and National Health Screening Programs to assess preeclampsia, low birth weight (LBW), and preterm delivery as risk factors for CVD. Cox proportional hazards models was used to evaluate the risk of total CVD, ischemic heart disease (IHD), and stroke after the pregnancy complications, with adjustment for potential confounding variables.

**Results:**

Women with CVD were likely to have a higher prevalence of pregnancy complications than women without CVD. The risk of total CVD was associated with preeclampsia (adjusted hazard ratio (HR), 1.60 [95% confidence interval (CI) 1.50–1.72]), LBW (1.20 [1.12–1.28]), and preterm delivery (1.32 [1.22–1.42]), after adjustment for confounders, including cardiovascular risk factors before pregnancy. The risk estimates of pregnancy complications for IHD were higher than those for stroke. In this study, the risk of total CVD was higher in the combined presence of preeclampsia and preterm delivery (2.23 [1.57–3.17] or all three complications (2.06 [1.76–2.40]), relative to no complications. The highest HR was noted in the risk of all pregnancy complications for IHD (2.39 [1.98–2.89]).

**Conclusion:**

Preeclampsia, preterm delivery, and LBW were independently associated with CVD in young Korean women. In addition, the combination of pregnancy complications had less-than-additive effects on CVD incidence.

**Supplementary Information:**

The online version contains supplementary material available at 10.1186/s12884-022-04578-2.

## Background

Cardiovascular disease (CVD) is one of the leading causes of death in South Korea and worldwide. Although the mortality rate of CVD is decreasing in some developed countries [1–3], the mortality rate of ischemic heart disease (IHD) per 100,000 population in South Korea has increased [4]. Moreover, cardiovascular morbidity and mortality rates in young women have increased even in developed countries [2, 5]. Due to the relatively lower incidence of CVD in young women, limited research is available that identifies this population as a vulnerable but underestimated group with worsening cardiac risk profiles [5–8]. Furthermore, prior research has shown a sex difference in mortality rate of IHD among women aged less than 55 years, and this difference is not fully explained by traditional cardiac risk factors [5, 9, 10]. Therefore, identification of young women who are at a high risk of CVD is of principal importance to improve women’s health. 

Pregnancy complications, which may affect young women to a greater extent, have been proposed as sex -specific risk factors of CVD, and have been identified in the latest guidelines on the prevention of CVD in many countries [11–14]. Pregnancy is associated with a profound maternal cardiovascular adaptation, and maladaptation during pregnancy is often suggested as a mechanism of pregnancy- induced complications [15]. Most of these changes revert back to normal after delivery; however, some women remained at increased risk of developing CVD, such as myocardial infarction, venous thromboembolism, and stroke, at later stages in life [16]. Many studies have shown that women with a history of preeclampsia, gestational diabetes, preterm delivery, and delivery of low birth weight (LBW) infants have an increased risk of cardiometabolic risk factors and subsequent CVD [3, 15, 17–20]. However, most studies have focused on older women because the incidence of CVD tends to increase with age. Despite the increase in the incidence of diabetes and hypertension due to the rise in the prevalence of obesity and adverse lifestyle factors such as sedentary lifestyle among young women, only few studies are available on the association between pregnancy complications and CVD outcome in young women. Furthermore, since most studies have focused on CVD outcomes in Westerners, studies on Asian women are lacking. In addition, only few studies have investigated the combination of pregnancy complications that confer a higher risk and whether they predict CVD at later stages in life within 10 years of pregnancy.

Therefore, we used nationally representative data to (1) compare obstetric outcomes in Korean women with and without future CVD within 10 years after pregnancy, (2) assess whether pregnancy complications are independent risk factors, and (3) investigate the combination of pregnancy complications as an additive function of determining risk factors for CVD.

## Methods

### Data source

This study was conducted by combining the databases of the Korea National Health Insurance Service (NHIS) claims, National Health Screening Examination (NHSE), and National Health Screening Program for Infants and Children (NHSP-IC) in South Korea. The study was approved by the Institutional Review Board of Korea University (2021GR0007). In South Korea, the NHIS is the only insurance provider, and 98% of the population is enrolled in this program. The database of NHIS claims contains almost all information, including anonymous personal information, health examination data, diagnoses, treatments, and hospitalizations, for each individual. All participants are required to undergo a biannual standardized health examination (NHSE) by the Korean government. The NHSE consists of a health interview and health examination. Details about pre-pregnancy factors were obtained using the NHSE database. The NHSP-IC requires all neonates to undergo seven consecutive health examinations at defined age groups (4–9, 9–18, 18–30, 30–42, 42–54, 54–66, and 66–80 months). The NHSP-IC has two components: a health interview with the parents and a health examination of their children. Information on preterm birth and birth weight was obtained through an NHSP-IC health interview.

### Study population

**Fig. 1:**
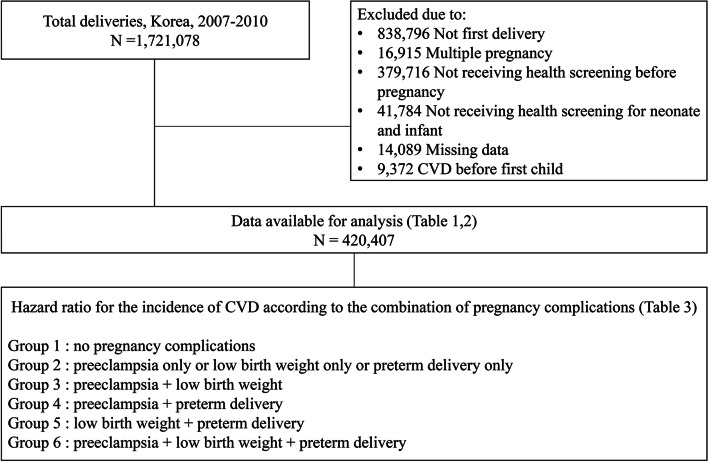
Flow diagram of number of study populations

This study included 1,721,078 women who delivered their babies between January 1, 2007, and December 31, 2010 (Fig. 1). Multiparous women (*n* = 838,796), those with multiple pregnancies (*n* = 16,914), those who did not undergo health examination before pregnancy (*n* = 379,716) or NHSP-IC for their babies (*n* = 41,784), those who already had CVD before pregnancy (*n* = 9,372), and those with missing data (*n* = 14,089) were excluded. In total, 420,407 women were eligible for this analysis, and their CVDs until 2018 were evaluated. In addition, to determine the incidence of CVD according to the combinations of pregnancy complications (preeclampsia, preterm delivery, and LBW), the groups were subdivided into six groups as follows: no complications, preeclampsia only or LBW only or preterm delivery only, preeclampsia + LBW, preeclampsia + preterm delivery, LBW + preterm delivery, and preeclampsia + LBW + preterm delivery**.**

### Outcomes

Using the database of NHIS claims, CVD was identified based on primary or secondary diagnosis according to the International Classification of Diseases 10th Revision (ICD-10) codes (Supplement). Women were classified as having CVD if they were diagnosed with CVD between delivery and December 31, 2018, wherein CVD comprised IHD and stroke.

### Covariates

Obstetric and baseline characteristics before pregnancy were identified using the dataset. Available information included age at delivery, delivery mode, birth weight, and preterm delivery. Women with gestational diabetes, preeclampsia, placental abruption, and placenta previa were identified using ICD-10 codes. Preterm birth was defined as gestational age of < 37 weeks, LBW was defined as birth weight of < 2.5 kg, and macrosomia was defined as birth weight of > 4.0 kg. Preeclampsia was defined using ICD codes for gestational hypertension, preeclampsia, superimposed preeclampsia, and eclampsia. Baseline characteristics before pregnancy were obtained from the NHSE database. Data for the following covariates were obtained from the health interview: previously diagnosed hypertension, diabetes, and smoking status. The health examination included calculation of body mass index (BMI), which was measured as weight in kilograms divided by measured height in meters squared. Blood pressure (BP) was measured using a standard mercury sphygmomanometer. Blood samples were obtained after fasting for at least 8 h. The levels of aspartate aminotransferase (AST), alanine aminotransferase (ALT), fasting glucose, and total cholesterol were measured using enzymatic methods.

### Statistical analysis

Continuous and categorical variables are presented as mean ± standard deviation and percentages, respectively. Baseline characteristics were compared between women with CVD and women without CVD using the t-test for continuous variables and chi-square test for categorical variables. The Cox proportional hazards models were used to estimate the adjusted hazard ratios (HRs) at 95% confidence intervals (CIs) for the development of CVD after pregnancy complications. For multivariable analyses, we adjusted for maternal age, BMI, systolic BP, diastolic BP, AST level, ALT level, fasting glucose level, total cholesterol level, and smoking. We also evaluated the risk of CVD according to the combination of pregnancy complications. The significance level was defined as a p-value of < 0.05. Statistical analyses were performed using SAS for Windows version 9.4 (SAS Inc., Cary, NC, USA).

## Results

The study population included 420,407 women. Of these, 5.29% of women (22,253) developed CVD events during the study period, including 12,672 IHD and 10,718 stroke cases. Characteristics of the study participants are presented in Table 1, with a comparison between women with and without CVD. Regarding obstetric characteristics, women with CVD were older at delivery and had a higher percentage of pregnancy complications (preterm delivery, LBW, and preeclampsia) than women without CVD. In addition, women with CVD were more likely to have a higher prevalence of cardiovascular risk factors before pregnancy (high BMI, hypertension, abnormal liver enzyme level, high fasting glucose level, high total cholesterol level, and current smoking) than women without CVD.

**Table 1:** Baseline characteristics of the study population

	Women without CVD (*n* = 398,154)	Women with CVD (*n* = 22,253)	*P*-value
Obstetric characteristics			
Age at delivery, y	29.37 ± 3.06	29.70 ± 3.39	< 0.001
Age at delivery ≥ 35	23,180 (5.82)	1,849 (8.31)	< 0.001
Cesarean delivery	128,851 (32.36)	8,361 (37.57)	< 0.001
Preterm delivery	10,427 (2.62)	785 (3.53)	< 0.001
Birth weight, kg	3.21 ± 0.47	3.20 ± 0.49	0.005
Low birth weight	14,465 (3.63)	977 (4.39)	< 0.001
Macrosomia	14,612 (3.67)	888 (3.99)	0.014
Male fetus	204,762 (51.43)	11,382 (51.15)	0.417
Gestational diabetes	5,212 (1.31)	400 (1.80)	< 0.001
Preeclampsia	8,616 (2.16)	819 (3.68)	< 0.001
Placenta abruption	1,777 (0.45)	119 (0.53)	0.055
Placenta previa	3,669 (0.92)	253 (1.14)	0.001
Baseline characteristics before pregnancy			
BMI, Kg/m^2^	20.59 ± 3.75	20.84 ± 2.83	< 0.001
BMI ≥ 25 kg/m^2^	23,422 (5.88)	1,796 (8.07)	< 0.001
Systolic BP, mm Hg	110.81 ± 11.03	111.59 ± 11.68	< 0.001
Diastolic BP, mm Hg	69.82 ± 8.27	70.43 ± 8.69	< 0.001
BP ≥ 140/90 mm Hg	8,178 (2.05)	676 (3.04)	< 0.001
Hypertension	15,949 (4.01)	1,455 (6.54)	< 0.001
AST, U/L	19.43 ± 11.40	19.85 ± 10.10	< 0.001
ALT, U/L	15.20 ± 16.46	15.91 ± 16.73	< 0.001
Abnormal liver enzyme	19,975 (5.02)	1,343 (6.04)	< 0.001
Fasting glucose, mg/dL	86.29 ± 12.95	86.61 ± 16.61	0.005
Fasting glucose ≥ 126 mg/dL	1,994 (0.50)	171 (0.70)	< 0.001
Diabetes	13,444 (3.38)	1,138 (5.11)	< 0.001
Total cholesterol, mg/dL	173.71 ± 31.92	175.28 ± 32.06	< 0.001
Total cholesterol ≥ 200 mg/dL	68,247 (17.14)	4,248 (19.09)	< 0.001
Smoking			< 0.001
Non-smoker	371,493 (93.30)	20,477 (5.22)	
Ex-smoker	11,902 (2.99)	805 (3.62)	
Current smoker	14,759 (3.71)	971 (4.36)	

*ALT* alanine transaminase, *AST* aspartate transaminase, *CVD* cardiovascular disease, *BMI* body mass index, *BP* blood pressure

Values are expressed as mean ± standard deviation or as n (%)

Table 2 presents the results of the Cox proportional HR of the effect of pregnancy complications on the subsequent incidence of total maternal CVD, stroke, and IHD. Women who experienced preeclampsia showed an approximately doubled risk of total CVD compared with women without preeclampsia, and this increased risk remained significant even after adjusting for potential confounders (HR, 1.60; 95% CI, 1.50–1.72). The risk of total CVD was 1.20-fold (95% CI 1.12–1.28) for LBW and 1.32- fold (95% CI 1.25–1.44) for preterm delivery. The risk estimates of pregnancy complications were higher for IHD than for stroke.

**Table 2:** Cox proportional hazard ratio of the association between pregnancy complications and subsequent maternal CVD

	**Unadjusted HR**		**Adjusted HR**^**a**^	
	HR	95% CI	HR	95% CI
Total CVD				
Preeclampsia	1.70	1.58, 1.82	1.60	1.50, 1.72
Low birth weight	1.23	1.15, 1.31	1.20	1.12, 1.28
Preterm delivery	1.37	1.27, 1.47	1.32	1.23, 1.42
IHD				
Preeclampsia	1.79	1.63, 1.96	1.66	1.51, 1.81
Low birth weight	1.30	1.19, 1.41	1.26	1.16, 1.37
Preterm delivery	1.43	1.30, 1.57	1.38	1.26, 1.51
Stroke				
Preeclampsia	1.61	1.45, 1.79	1.54	1.39, 1.71
Low birth weight	1.17	1.07, 1.29	1.15	1.05, 1.26
Preterm delivery	1.33	1.20, 1.48	1.30	1.17, 1.44

*CI* confidence interval, *CVD* cardiovascular disease, *HR* hazard ratio, *IHD* ischemic heart disease

^a^Adjusted for maternal age, BMI, systolic BP, diastolic BP, aspartate aminotransferase, alanine aminotransferase, fasting glucose, total cholesterol, and smoking

Table 3 presents the association between combinations of pregnancy complication (preeclampsia, preterm delivery, and LBW) and the subsequent risk of CVD. For women who had preeclampsia with other complications, the adjusted HR for future CVD was 1.29 (1.00–1.67) with LBW and 2.23 (1.57–3.17) with preterm delivery, as compared with women who had neither. Women who experienced all three pregnancy complications were twice as likely to develop subsequent CVD than were women who experienced none of the complications (HR, 2.06; 95% CI, 1.76–2.40). A similar significant effect was noted in the subsequent development of IHD. Moreover, the risk of stroke increased with the addition of pregnancy complications; however, these increases were minimal. The highest HR was noted in the risk of all pregnancy complications for IHD (HR, 2.39; 95% CI, 1.98–2.89).

**Table 3:** Cox proportional hazard ratio of the association between combinations of pregnancy complications and subsequent maternal CVD

	**Unadjusted HR**		**Adjusted HR**^**a**^	
	HR	95% CI	HR	95% CI
Total CVD				
No complications	1		1	
PE or LBW or PTD	1.31	1.23, 1.38	1.27	1.20, 1.35
PE + LBW	1.38	1.07, 1.79	1.29	1.00, 1.67
PE + PTD	2.46	1.73, 3.49	2.23	1.57, 3.17
LBW + PTD	1.22	1.10, 1.36	1.19	1.07, 1.33
PE + LBW + PTD	2.21	1.89, 2.58	2.06	1.76, 2.40
IHD				
No complications	1		1	
PE or LBW or PTD	1.33	1.24, 1.43	1.28	1.20, 1.38
PE + LBW	1.50	1.08, 2.09	1.38	0.99, 1.91
PE + PTD	2.57	1.64, 4.04	2.28	1.45, 3.58
LBW + PTD	1.25	1.08, 1.44	1.21	1.04, 1.39
PE + LBW + PTD	2.62	2.17, 3.16	2.39	1.98, 2.89
Stroke				
No complications	1	1	1	
PE or LBW or PTD	1.27	1.17, 1.38	1.25	1.15, 1.35
PE + LBW	1.33	0.08, 1.93	1.26	0.86, 1.83
PE + PTD	2.90	1.83, 4.61	2.70	1.70, 4.29
LBW + PTD	1.26	1.08, 1.47	1.23	1.05, 1.43
PE + LBW + PTD	1.74	1.35, 2.23	1.64	1.28, 2.10

*CI* confidence interval, *CVD* cardiovascular disease, *HR* hazard ratio, *PE* preeclampsia, *LBW* low birth weight, *PTD* preterm delivery, *IHD* ischemic heart disease

^a^Adjusted for maternal age, BMI, systolic BP, diastolic BP, aspartate aminotransferase, alanine aminotransferase, fasting glucose, total cholesterol, and smoking

## Discussion

This study demonstrated that women with CVD had higher cardiovascular risks before pregnancy and a higher prevalence of pregnancy complications and that preeclampsia, preterm delivery, and LBW were independent risk factors for CVD, even after adjustment for confounders. The combination of pregnancy complications had less-than-additive effects on CVD incidence.

Our study findings are similar to those of previous studies that linked pregnancy complications with subsequent CVD. Many epidemiological studies and meta-analyses have reported an association between preeclampsia and an increased risk of future CVD [3, 18, 21, 22]. Depending on the study, the risk estimates varied from 1.78 to 2.28-fold for total CVD [17, 18], 2.16- to 2.50-fold for IHD [17, 22], and 1.65- to 1.81-fold for stroke [17, 18, 22]. The risk of CVD was increased in many studies that included women who delivered a neonate with LBW. Across various studies, women with LBW infants have been found to be approximately twice as likely to develop CVD in the future [3, 23]. Other studies suggest a 1.2- to 2.5-fold increased risk of CVD in women with a history of preterm delivery [19, 23–25]. Preterm delivery remains associated with CVD even in pregnancies not complicated by preeclampsia or LBW/small-for-gestational age (SGA) [19, 25, 26]. Our study reported similar risk levels.

However, whether there is an additive effect on the incidence of cardiovascular events when experiencing combinations of these complications is still debated. A previous study showed that the risk of IHD with preeclampsia alone was 1.82 and that the risk was slightly increased to 2.26 when all three pregnancy complications were combined, which is similar to our results [20]. However, other studies have shown that the combination effect was dose-dependent. In a recent study, the HR for major coronary events was 2.1 after preeclampsia alone, 3.3 after preeclampsia in combination with SGA, and 5.4 after preeclampsia in combination with preterm delivery [27]. Other studies have also demonstrated that women with all three complications had a 7 times higher risk of IHD-related hospital admission or death than those in without any complications [23, 24].

The observed discordance among these previous studies could have resulted from heterogeneity in the background of the study population and the final outcome measures. Previous studies differ from our study in having a follow-up period of more than 15 years and including a wide range of women up to a relatively older age [23, 24, 27]. The final outcome included not only CVD incidence but also CVD mortality rate [23, 28]. Only one study has demonstrated results similar to ours, and it also included only young women who were followed up for only 10 years after delivery [20]. Different characteristics of women depending on follow-up period could affect the results concerning the relationship between pregnancy complications and CVD, in that 10-year and 20-year CVD prediction models showed different discrimination when incorporating preterm delivery and parity into the models in a recent study [29].

Another explanation for the discordance could be racial differences. Previous studies have shown that racial/ethnic differences affect the incidence and mortality rate of CVD [30, 31]. Most previous studies were limited to Caucasian races as the study population. Only one study from Taiwan showed that women with a history of preeclampsia/eclampsia had an increased risk of stroke, but the number of stroke cases was limited [32]. To the best of our knowledge, no study to date has examined the relationship between pregnancy complications and overall CVD in young Asian women. Given the possible effects of race on CVD incidence, further studies are needed to investigate the different effects of race on CVD after pregnancy.

It is unclear whether pregnancy complications have independent effects on future CVD, an early marker of women with preexisting high-risk profiles for future CVD, or both. A recent study showed that women with preterm delivery had an adverse cardiovascular risk factor profile before pregnancy [33], whereas another study reported no evidence of an increased risk being explained by conventional risk factors in women with preterm delivery [34]. In this study, we showed the independent effects of pregnancy complications on future CVD and demonstrated that women with CVD already had risk factors before pregnancy. Therefore, we speculate that pregnancy complications have independent effects on future CVD, but the contribution to CVD of a pregnancy complication could be smaller than the contribution of conventional risk factors that were present before pregnancy. Furthermore, Romundtad showed that the association between preeclampsia and cardiovascular risk factors after pregnancy was substantially attenuated after adjustment for pre-pregnancy risk factors and reported that cardiovascular risk factors present before pregnancy are more important determinants of subsequent cardiovascular risk factors than pregnancy complications [35]. In the study of clinical utility pregnancy complications in 10-year CVD risk prediction, additional inclusion of pregnancy complications in an established risk score did not improve the discrimination of high-risk populations [36, 37].

Another important finding of our study is that the risk estimates of pregnancy complications were higher for IHD than for stroke; this finding is consistent with the findings of previous studies [17, 38]. A drastic decline in the mortality rate of stroke over the past decades was observed in both the sexes and across all races and age groups, and these significant improvements are thought to result from conventional cardiovascular risk factor control interventions [2]. Despite these interventions, the mortality rate of IHD in South Korea is still increasing, whereas the mortality rate in the United States did not decrease as much [2, 4]. Compared with traditional risk factors, we assumed that other factors, such as complications during pregnancy, may have a greater effect on the occurrence of IHD than of stroke.

Our study had some limitations. First, although we adjusted for some risk factors in the analysis, we may have inadequately accounted for socioeconomic, dietary, and lifestyle confounding factors that are known to affect both pregnancy complications and CVD. This data was not available in the registries that we used. Second, diagnosis misclassification and under-reporting of diseases existed for ICD-10 codes. Third, there were no detailed data on pregnancy, such as the severity of preeclampsia/eclampsia or LBW, early or late preterm delivery, and whether these women received treatment during the follow-up period.

The strengths of our study include the large sample size, the prospectively collected data, and the non-differential follow-up of CVD incidence in nationwide registers. Moreover, inclusion of only primiparous women giving birth over the full range of gestational ages from 20 weeks onward adds to the strengths of this study.

Evidence shows that after guidelines are followed and risk factors are appropriately addressed and managed, the mortality rate of CVD decreases by a third and the outcomes improve [7, 8, 16]. Therefore, guidelines for recognizing pregnancy complications in clinical practice could identify high-risk women in advance and potentially benefit public health through early preventive strategies.

## Supplementary Information

**Additional file 1:Table S1.** International classification of disease codes used in the analysis.

## Data Availability

All data generated or analyzed during this study are included in this published article.

## Abbreviations

ALT: Alanine aminotransferase
AST: Aspartate aminotransferase
BMI: Body mass index
BP: Blood pressure
CI: Confidence intervals
CVD: Cardiovascular disease
HR: Hazard ratio
ICD: International Classification of Diseases
IHD: Ischemic heart disease
LBW: Low birth weight
NHIS: National Health Insurance Service
NHSE: National Health Screening Examination
NHSP-IC: National Health Screening Program for Infants and Children
SGA: Small-for-gestational age
