# Supplementary material for: Prior pregnancy complications and maternal cardiovascular disease in young Korean women within 10 years after pregnancy
Source: BMC Pregnancy Childbirth. 2022 Mar 21;22:229. doi: 10.1186/s12884-022-04578-2 (PMC8935765; doi:10.1186/s12884-022-04578-2)
Supplement: Supplementary file 1 — Additional file 1:Table S1. International classification of disease codes used in the analysis. [file 12884_2022_4578_MOESM1_ESM.docx]

**Table S1: International classification of disease codes used in the analysis**

| Diagnosis | ICD-10 Codes |
| --- | --- |
| Cardiovascular disease |  |
| Ischemic heart disease | I20.x-I25.x |
| Stroke | I60.x-I69.x |
| Gestational diabetes | O24.4, O24.9 |
| Preeclampsia | O13-O16 |
| Placental abruption | O45 |
| Placenta previa | O44 |

*ICD-10* International Classification of Diseases, 10th Revisions
